# Telekit: a bilateral, teleoperated platform for hands-on learning

**DOI:** 10.3389/frobt.2026.1734211

**Published:** 2026-04-07

**Authors:** Chuyuan Bie, Irene A. Kuling, Julie Legrand

**Affiliations:** 1 Department of Mechanical Engineering, Eindhoven University of Technology, Eindhoven, Netherlands; 2 Eindhoven Artificial Intelligence Systems Institute, Eindhoven University of Technology, Eindhoven, Netherlands

**Keywords:** bilateral teleoperation device, control, design, haptics education, kinesthetic devices

## Abstract

**Introduction:**

Bilateral teleoperation enables intuitive interaction with remote environments and is widely used in surgery, space, and industry. However, educational tools often rely on costly hardware or purely virtual setups, limiting accessibility and reducing opportunities for hands-on learning. This paper presents the Telekit, a low-cost bilateral teleoperation system designed to support practical education.

**Methods:**

The Telekit is built on Stanford’s open-source Hapkit and features a gear-based transmission for improved robustness, along with a force-sensitive resistor at the handle for rendering real-world interactions. Communication and control are implemented using MATLAB, achieving a measured single-byte round-trip delay of 2 ms. Two control strategies, Position–Position (PP) and Position–Force (PF), were developed and tested in both virtual and physical environments. PP control incorporates damping and friction compensation, while PF control utilizes force feedback from the integrated sensor.

**Results:**

For PP control at low frequency (0.24 Hz), damping combined with friction compensation resulted in a mean communication delay of 230 ms and a mean tracking error of 3.4 ° between leader and follower units. At higher frequency (1.15 Hz), damping alone reduced the mean communication delay to 140 ms but increased the mean tracking error to 8.7 ° . PF control enabled users to perceive different stiffness levels, with soft, medium, and hard stiffness measured at 0.17 N/ ° , 0.41 N/ ° , and 0.68 N/ ° , respectively.

**Discussion:**

The Telekit demonstrates that a low-cost platform can effectively support both position tracking and stiffness perception. Although performance varies with operating frequency, the system provides meaningful haptic feedback and reliable functionality. As such, it offers an accessible, hands-on solution that bridges theoretical concepts and practical experimentation in teleoperation education.

## 1 Introduction

Bilateral teleoperation significantly improved interactions between humans and remote environments over the past 2 decades. This technology allows an operator to remotely control a robot or manipulator. When used with sensors, bilateral teleoperation enables the operator to receive force feedback from the environment, which makes it particularly effective for complex and precise tasks in hazardous, inaccessible, or remote scenarios. Applications of bilateral teleoperation are widely used in fields such as robotic surgery, space exploration, and industrial maintenance (Hokayem and Spong, 2006). Therefore, the development of an educational tool for bilateral teleoperation is crucial for providing future engineers with hands-on experience in control systems and robotics. Such tools bridge theoretical knowledge in robotics and teleoperation with practical applications, ensuring a deeper understanding of the field.

Previous studies explored the development of educational tools for teleoperation and robotic systems by leveraging internet-based platforms to deliver cost-effective remote control solutions in educational settings (Candelas et al., 2003; Kaarlela et al., 2022; Pala et al., 2012; Yang et al., 2004). These approaches primarily relied on virtual simulations, offering low-cost environments that facilitated conceptual understanding. While such approaches provide accessible learning environments, they generally lack direct physical interaction with real hardware systems. However, hands-on experience was shown to significantly enhance student learning outcomes and skill acquisition in engineering education (Hofstein and Lunetta, 2004).

To address the need for physical interaction, Rojko et al. introduced a motion control and teleoperation challenge featuring a hardware follower platform (Rojko and Hercog, 2015), while Koenig et al. developed a gesture-based teleoperated grasping robot using leap motion’s optical gesture tracker to control a manipulator (Koenig et al., 2021). Although both systems enable remote control, they do not include a physical master device, which limits their ability to deliver true bilateral interaction with force feedback. Another notable contribution was Marinho et al.‘s UMIRobot, an open-source, low-cost robotic manipulator equipped with six servo motors (Marinho et al., 2023). Although the system is affordable and flexible, controlling six motors makes it complex, which might make it harder for students to learn the basic ideas of bilateral teleoperation. Overall, existing educational platforms either rely primarily on virtual environments or involve hardware configurations that remain relatively complex for introductory teaching contexts.

Some research also focused on high-cost, high-performance teleoperation platforms (Beerens et al., 2019; Nuño et al., 2009; Yang et al., 2015). While these systems are valuable for advanced research, their technical complexity and financial requirements limit their adoption in educational contexts. Consequently, a gap remains between simple virtual teaching tools and complex research-grade teleoperation platforms. As a result, there is a clear need for a low-cost, physically interactive bilateral teleoperation platform that enables students to gain hands-on experience with force-feedback control, without the barriers posed by complex or expensive systems.

This paper presents the Telekit, a low-cost bilateral teleoperation system for education, built on the Stanford University’s open-source Hapkit haptic device (Martinez et al., 2019). The Hapkit is a one-degree-of-freedom kinesthetic haptic device that enables users to input motions and experience programmed forces, designed for hands-on laboratory experiences. As an open-access platform, both in hardware and software, it was widely adopted by universities worldwide to teach haptics, including TU Delft (Netherlands) (Dijkstra et al., 2014) and Columbia University (United States) (Okamura et al., 2014), with each institution developing its own adaptations. While current versions of the Hapkit support interaction with virtual environments, they do not yet enable bilateral teleoperation with physical hardware systems. This limitation highlights the absence of a simple and affordable platform enabling students to experiment with both motion control and force feedback in a bilateral teleoperation setup. The Telekit is specifically designed as an extension to the existing Hapkits, enabling seamless integration into the current Hapkit-based educational program.

This paper therefore introduces a low-cost bilateral teleoperation platform specifically designed for education, the Telekit. The main contribution of this work is the development of an accessible hardware and software platform that enables students to experiment with bilateral teleoperation, including both position and force feedback interactions. The system’s design is tailored for student testing, with software developed to enable Position-Position (PP) and Position-Force (PF) control. Additionally, the controllers have been fine-tuned to enhance the Telekit performances, and those performances are analyzed and discussed.

## 2 Materials and methods

### 2.1 Hardware

The design was based on the original Hapkit developed by Stanford University, with adaptations made specifically to maximize robustness during repeated student testing. The friction wheel mechanism used in Hapkit 2.0 to transmit motor torque to the system handle was prone to issues such as insufficient grip due to its sensitivity to wheel misalignment. Additionally, the capstan drive mechanism in Hapkit 3.0 was not robust enough for repeated student use, often breaking when the motor exceeded its intended position (Martinez et al., 2019). As a result, a spur gear system was selected as the transmission method for the teleoperation hardware, providing more reliable actuation without the risk of slippage or over-rotation. In the current prototype, the handle was laser-cut from plexiglas and the transmission wheel was 3D-printed in PLA. The inherent tolerances of the 3D-printed gear could introduce minor transmission errors and reduce smoothness. Consequently, the actuation was sensitive to manufacturing deviations in the gear system, which might have resulted in less fluid operation.

**FIGURE 1:**
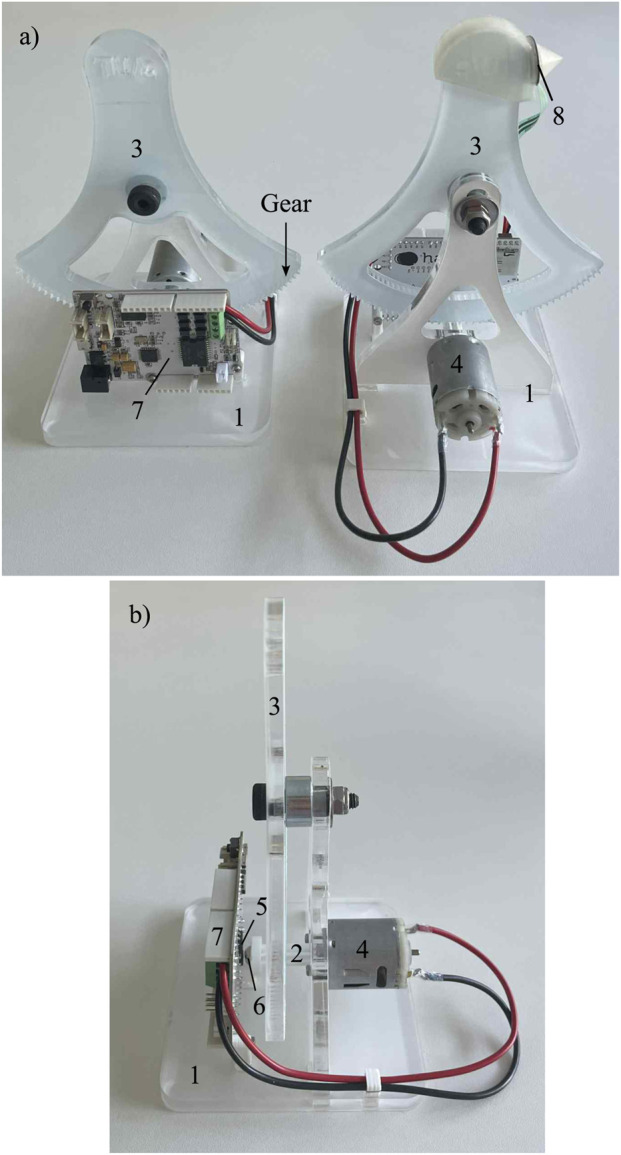
Overview of the Telekit hardware. **(a)** front and back view; **(b)** side view. 1. frame; 2. transmission wheel; 3. handle; 4. motor; 5. magnetoresistive sensor; 6. magnet; 7. Hapkit board (Seeed Studio); 8. force-sensitive resistor.

Both leader and follower teleoperated interfaces consisted of three primary components: a plexiglas frame, a 3D-printed PLA transmission wheel, and a plexiglas handle (Figure 1). A 12-V, 5600 RPM motor (MOT3N, Velleman, Belgium) was mounted onto the frame and was connected to the transmission wheel by securing its shaft inside the wheel with adhesive. For position measurement, a magnetoresistive sensor (KMA310, NXP, the Netherlands) detected the angular position of a 6 mm cylindrical magnet attached to the transmission wheel, on the side opposite the motor shaft. The motor and sensor were controlled using the Hapkit board from Seeed Studio. Additionally, a force-sensitive resistor (FSR402) was attached to a 3D-printed bracket and was mounted on the handle of the follower interface to measure environmental forces.

The total hardware cost of one Telekit unit was approximately 60 €, which was significantly lower than commercial haptic devices such as the Geomagic Touch/Phantom Omni (priced in the several thousand euro range) or the Novint Falcon ( ∼ 250 $). This cost difference made the Telekit more accessible for classroom use and for deploying multiple units in educational labs.

### 2.2 Communication system

Efficient communication between the leader and follower interfaces was crucial for real-time bilateral teleoperation. The quality of communication directly impacted the delay and stability of transmitted data. To achieve reliable and high-speed data exchange, various communication methods were tested, including USB-TTL, USB cables, and Dupont cables, using both hardware and software communication approaches.

#### 2.2.1 Dupont cable for hardware communication

Hardware communication between the two Hapkit boards was initially established using Dupont wires by directly connecting the TX pin of one board to the RX pin of the other, and *vice versa*, with a common ground. Although this configuration removed the need for intermediary software and was theoretically capable of low-latency transfer, Dupont wires were unshielded and susceptible to electrical noise and mechanical instability. These factors resulted in inconsistent signal quality and occasional packet loss. Moreover, relying solely on this hardware link restricted data acquisition, ultimately introducing instability and delays that hindered system performance.

#### 2.2.2 Dupont cable for software communication

A second approach used the Arduino SoftwareSerial library to reassign communication pins while still relying on Dupont cables. In practice, this method proved unsuitable for real-time control. Tests revealed substantial latency variations and frequent transmission failures, making this approach impractical for stable bilateral teleoperation.

#### 2.2.3 USB cable for hardware communication

The chosen implementation used USB cables for hardware communication, with MATLAB (MathWorks) acting as an intermediary for data management. This approach combined the speed and reliability of hardware communication with the flexibility of software for data recording and analysis. USB communication provided shielded, differential signaling, offering higher signal integrity and more consistent latency than Dupont-based approaches. To assess the communication delay of this communication method, single-byte transmission tests were conducted, simulating the leader-to-follower communication process. The average round-trip delay was approximately 2 ms, well within the 20 ms threshold for real-time requirements (Millnert, 2014). This low latency ensured fast and stable transmission of control and feedback data, enabling responsive and reliable bilateral teleoperation.

Regarding position data transmission between the leader and follower devices, floating-point values were converted to 2-byte integers. This approach reduced bandwidth usage, increased data transfer speed, and minimized latency, meeting the needs of real-time control systems (Marwedel, 2021).

### 2.3 Controller implementations

A PP and a PF controller were implemented to allow bidirectional position synchronization and haptic feedback, enabling users to both control and physically sense remote interactions in real or simulated environments. The PP controller was further enhanced by incorporating additional damping to mitigate instability from communication delays and friction compensation to counteract internal resistance from the motor and transmission at low speed.

#### 2.3.1 Position-position controller

The primary objective of the PP controller was to achieve motion synchronization between the leader and follower devices, primarily through the real-time transmission of position and velocity data. The controller relied on proportional (P) and derivative (D) control strategies to ensure the slave device accurately replicated the leader’s movements.

The dynamics of the leader and follower devices are modeled as follows (Equations 1, 2):

$$M_{l}{\ddot{x}}_{l}+B_{l}{\dot{x}}_{l}=f_{lc}+f_{o}$$ (1)

$$M_{f}{\ddot{x}}_{f}+B_{f}{\dot{x}}_{f}=f_{fc}-f_{e},$$ (2)

where $M_{l}$ and $M_{f}$ are the masses of the leader and follower devices, respectively, $B_{l}$ and $B_{f}$ represent the damping coefficients of the leader and follower devices, and $f_{o}$ and $f_{e}$ are the forces applied by the operator on the leader and by the environment on the follower, respectively.

The PP controller model is expressed as Equations 3, 4:

$$f_{lc}=K_{p}\left(x_{f}-x_{l}\right)+K_{d}\left({\dot{x}}_{f}-{\dot{x}}_{l}\right)$$ (3)

$$f_{fc}=K_{p}\left(x_{l}-x_{f}\right)+K_{d}\left({\dot{x}}_{l}-{\dot{x}}_{f}\right),$$ (4)

where $f_{lc}$ and $f_{fc}$ denote the commands to assign forces to the leader and follower motors by the leader and follower controllers, respectively. $K_{p}$ and $K_{d}$ are the proportional and derivative gains, respectively. $x_{l}$ and $x_{f}$ are the position of leader and follower handles in degrees.

**FIGURE 2:**
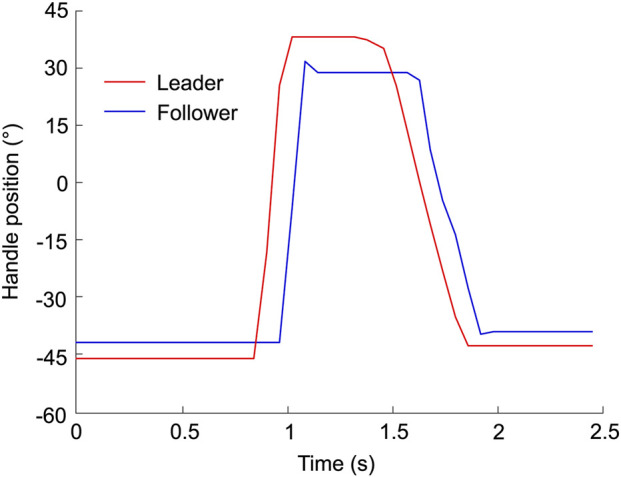
Leader and follower systems response to a step torque signal leading to a close to maximal handle position amplitude range. 0 ° corresponds to the handle vertical position.

The controller was initially tuned by applying a step torque signal to the leader device, resulting in a maximum handle position amplitude range. As shown in Figure 2, discrepancies between the leader and follower handle positions were observed, attributed to system delays and the motor’s drive threshold. The mean delay, as measured in Figure 2, is 120 ms, while the motor requires a torque exceeding 0.26 Nm, as experimentally determined, to overcome friction. Additionally, sensor inaccuracies contribute to the discrepancies between the leader and follower, as evidenced by the non-zero initial position of the device (Figure 2).

**FIGURE 3:**
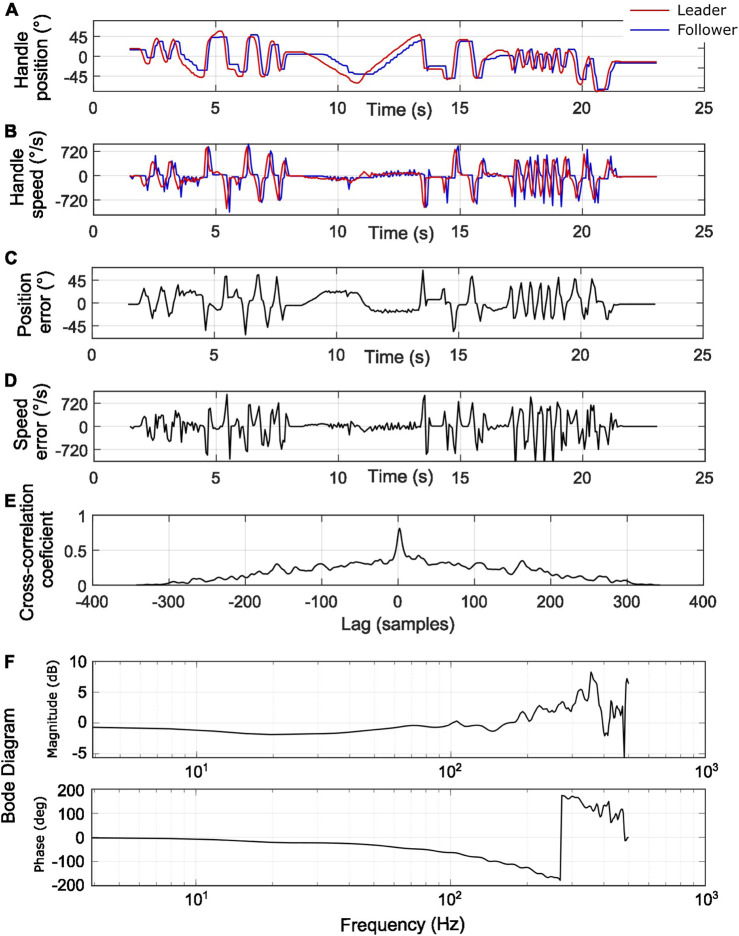
Leader and follower systems response to an arbitrary leader trajectory. **(A)** Handle positions; **(B)** Handle speeds; **(C)** Position error; **(D)** Speed error; **(E)** Cross-correlation coefficient; **(F)** Code diagram with Magnitude (up) and Phase (down).

Next, the control system was fine-tuned through applying a swept sine to the leader device motor, leading to the choice of parameters $K_{p}=0.21$ N/ ° and $K_{d}=0.0013$ Ns/ ° . Then, the controller was tested through manual operation, with the leader device handle moved arbitrarily starting from the vertical position. This resulted in a mean measured error of 15.3 ° for the handle position and a maximum error of 63.3 ° (Figure 3). In Figure 3, the cross-correlation coefficient quantifies the similarity between the leader and follower signals as a function of the time-lag between them. A peak value of 0.81 in the cross-correlation at 2 lag samples indicates a delay of 2 samples. Given the data acquisition frequency of 16.6 Hz, this corresponds to a delay of 120 ms. This peak suggests that most of the system errors are due to delays. After compensating for this delay in data processing, the error for the handle position improves to 9.3 ° , and the maximum error reduces to 20.16 ° . The Bode diagram of the system with the implemented PD controller (Figure 3F) shows that at low frequencies (0–10 Hz, i.e., in the frequency range relevant for human teleoperation), the magnitude remains between −0.7 and −1.2 dB, indicating mild attenuation, while the phase stays within −2 to −5°, reflecting delay. In the mid-frequency range (10–100 Hz), the magnitude reaches a minimum of −1.9 dB and the phase progressively decreases to approximately −63°, showing a more important delay. Above 100 Hz, the frequency response becomes dominated by noise and no longer represents the physical behavior of the system.

#### 2.3.2 Position-position controller with damping

To further reduce the error and ensure stable bilateral teleoperation of the system in the presence of time delays, a damping injection term was introduced in the PP control architecture. The damping term served several purposes: it stabilized the system by absorbing oscillatory energy caused by time delays and it ensured smoother control by reducing the jerky responses often caused by high proportional gains $\left(K_{p}\right)$ in delayed systems, improving interaction comfort.

The control laws were defined as follows (Equations 5, 6):

$$f_{lc}=K_{p}\left(x_{f}\left(t-T\right)-x_{l}\right)+K_{d}\left({\dot{x}}_{f}\left(t-T\right)-{\dot{x}}_{l}\right)-B{\dot{x}}_{l}$$ (5)

$$f_{fc}=K_{p}\left(x_{l}\left(t-T\right)-x_{f}\right)+K_{d}\left({\dot{x}}_{l}\left(t-T\right)-{\dot{x}}_{f}\right)-B{\dot{x}}_{f},$$ (6)

with t , the time, T , the constant time-delay, and $B{\dot{x}}_{l}$ and $B{\dot{x}}_{f}$ , the damping coefficients of the leader and follower devices, respectively. To ensure stability, the controller parameters needed to satisfy the following inequality (Equation 7) (Nuño et al., 2011; Lee and Spong, 2006):

$$2B^{2}>K_{p}^{2}T^{2}$$ (7)

Based on the actual measured communication delay of approximately 2 ms and the previously calculated proportional gain $K_{p}=0.21$ N/ ° , the damping coefficient B had to be greater than $3\cdot 10^{-4}$ Ns/ ° to ensure system stability. In the experiments, $B=3.21\cdot 10^{-4}$ Ns/ ° was selected.

#### 2.3.3 Compensation for friction-induced error

Another factor influencing the error between the leader and follower system was the motor’s drive threshold and the mechanical transmission between the handle and the motor. Specifically, for the motor to overcome its internal resistance and move the handle, the torque command had to exceed a threshold of 0.26 Nm, as measured experimentally. Especially at low speeds, the input torque was often too small to surpass the 0.26 Nm threshold, leading to position errors between the leader and the follower device. To mitigate this, a friction compensation term was added to the control force command to improve the devices’ responsiveness. An adaptive compensation term was introduced, adjusting the compensatory force based on the devices’ velocity feedback. This adaptive approach reduced the compensatory force as the devices’ velocity increased, maintaining stability while improving responsiveness. More precisely, the model was intended to compensate for the static and low-velocity friction regime without injecting excessive force at higher speeds. The compensation therefore decreased linearly with velocity magnitude, so that the largest correction was applied near zero velocity, where stick-slip effects were most pronounced, and progressively vanished as velocity increased.

The resulting control force f is given by Equations 8, 9:

$$f=f_{c}+XG$$ (8)

with:

$$G=sgn\left(\dot{x}\right)\cdot V_{\max}-\dot{x}$$ (9)

where $f_{c}$ is the force calculated by the PP controller, G is the adaptive force compensation, decreasing as the device velocity increases, X is a constant ensuring the adaptive force compensation term is always equal or smaller than the drive threshold of the system to ensure stability. $V_{\max}$ is the maximum velocity possible of the device handle and sgn() is the sign function. In the experiments, X=2.5 kg/s and $V_{\max}=933{}^{\circ}$ /s were selected.

This friction compensation method was particularly effective for unidirectional movements, especially over short distances and low speed movement, where the added compensation successfully overcame the initial static resistance without causing instability. However, for bidirectional or oscillatory motion and especially in the case of frequent direction changes, the system could experience lag due to adjustment of the compensating force direction.

#### 2.3.4 PF controller with damping and delay compensation

The PF control strategy in bilateral teleoperation enabled force reflection from a remote environment, allowing the operator to sense and interact with the environment through force feedback. In this configuration, the leader device acted as the virtual environment, while the follower mimicked the operator’s motions, generating forces based on environmental feedback. Based on the dynamics of the leader and follower devices, the mathematical model of the PF controller with damping and delay-compensation was expressed as follows (Equations 10, 11):

$$f_{lc}=-f_{e}-B_{l}{\dot{x}}_{l}+XG$$ (10)

$$f_{fc}=K_{p}\left(x_{l}-x_{f}\right)+K_{d}\left({\dot{x}}_{l}-{\dot{x}}_{f}\right)-B_{f}{\dot{x}}_{f}+XG$$ (11)

### 2.4 Statistical analysis

Tracking performance was quantified using the communication delay, the mean tracking error, and the maximum tracking error. For repeated sinusoidal tests, these quantities were computed for each trial and then summarized across trials using the mean and standard deviation. For the real-environment PF experiments, perceived stiffness values were calculated independently for each test and are reported as mean ± standard deviation over 10 trials per environment.

Delay was estimated using cross-correlation between leader and follower position signals. Tracking error was computed as the absolute position difference between the two signals, either directly or after delay compensation in post-processing, as specified in the corresponding subsection. For stiffness estimation, the ratio between measured follower force and leader displacement was computed over the contact phase of each trial.

All signal processing and statistical analyses were performed in MATLAB (MathWorks, Natick, MA, United States). Given the exploratory and proof-of-concept nature of the study, the analysis focused on descriptive statistics rather than formal hypothesis testing. Therefore, no inferential statistical significance tests were applied. Variability across repeated trials is reported using standard deviation, and representative trials are shown in the figures together with the corresponding average values across repetitions. Confidence intervals were not included because the primary aim was to characterize system behavior and feasibility rather than to test group-level hypotheses.

## 3 Results

### 3.1 PP controller

**FIGURE 4:**
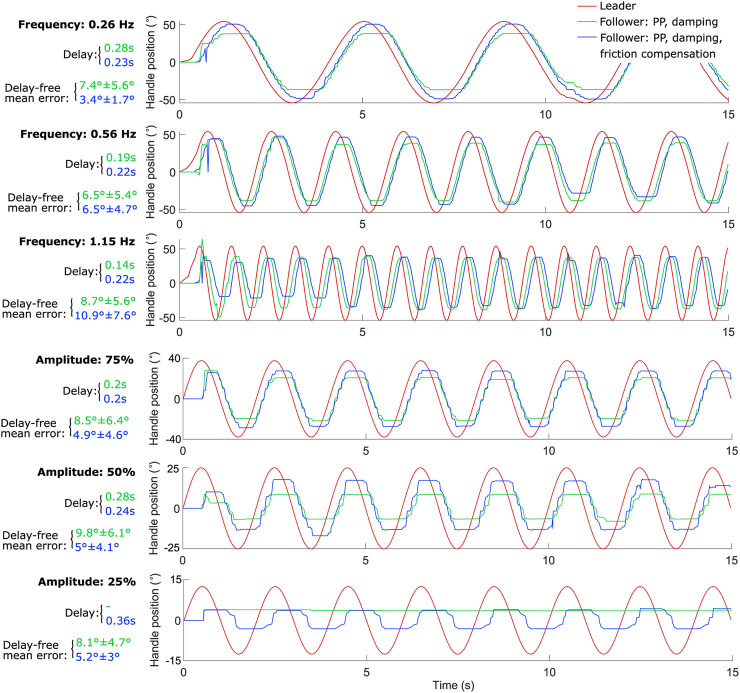
Handle positions of both the leader and follower units in response to a full-amplitude 0.26 Hz, 0.56 Hz, and 1.15 Hz sine wave, and a 0.5 Hz sine wave of 75%, 50% and 25% of the full-amplitude at the leader unit, using the implemented PP controller with added damping and friction-induced error compensation terms.

The performance of the PP controller with various add-ons, specifically damping and friction-induced error compensation, was evaluated by applying a full-amplitude sine wave to the leader device position signal at three different frequencies (0.26 Hz, 0.56 Hz, and 1.15 Hz), and three different amplitudes (75, 50% and 25% of the full amplitude) at 0.5 Hz. Figure 4 shows the corresponding follower device positions under two conditions: with damping only, and with both damping and friction-induced error compensation. Each test consisted of 15 cycles of the leader handle, with a 15-s segment shown in Figure 4. For each test, the measured communication delay as well as the mean and standard deviation of the tracking error were reported. The tracking error was defined as the absolute angular difference between the leader and follower handle positions. Two metrics were considered: (i) the raw tracking error, directly computed from the recorded signals, and (ii) the delay-free tracking error, computed after compensating for the measured communication delay by temporally shifting the follower signal using cross-correlation alignment. This procedure isolates the error due to control performance and mechanical effects from the delay-induced phase shift between the two devices.

As expected, the friction-induced error compensation had the greatest effect at lower frequencies (0.26 Hz), reducing both the delay and the delay-free tracking error compared to the setup without the friction compensation term. At the higher frequency of 1.15 Hz, however, the friction compensation term offered no noticeable improvement; on the contrary, it increased both the communication delay and the delay-free tracking error. This behavior was expected, as in bidirectional or oscillatory motion, particularly with frequent direction changes, the system might lag due to continuous adjustments in the direction of the compensating force.

It is important to distinguish between the measured communication delay and the overall system delay observed in the experimental signals. The communication delay measured at the byte transmission level was approximately 2 ms (see Methods Section). However, the effective system delay observed in the leader–follower motion responses reached approximately 120–230 ms. This larger delay results from the accumulation of several factors, including sensor acquisition time, signal processing in MATLAB, control loop execution and motor actuation dynamics. Consequently, the experimentally observed delay reflects the combined latency of the entire sensing–control–actuation loop rather than the communication channel alone.

The influence of the friction-induced error compensation term was particularly evident at lower amplitudes (Figure 4), where it helped overcome static friction during small master-side handle movements and frequent direction reversals. The error increased to 6.5, 10, and 20% for 75%, 50%, and 25% of the maximum master amplitude, respectively, when damping and friction compensation were implemented together. When only damping was implemented, it increased further to 11.2, 19.6, and 31.2%.

**FIGURE 5:**
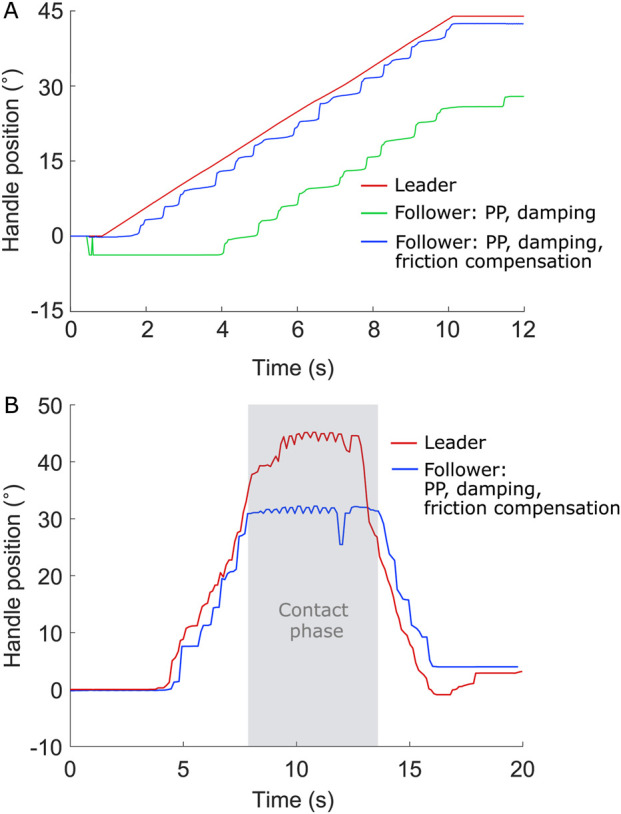
Handle positions of both the leader and follower units **(A)** in response to a 4.7 ° /s slope inputs at the leader unit. **(B)** when the follower unit makes contact with a hard surface.

To clearly demonstrate the effect of the friction-induced error compensation term, the leader handle position was programmed to follow a slow, linear trajectory with a slope of 4.7 ° /s, simulating a gradual start (Figure 5A). Under these conditions, the follower with friction-induced error compensation exhibited a mean tracking error of 2.4 ° , while the follower without that compensation showed a significantly larger mean error of 16.4 ° .

The system’s ability to render contact with the environment using a PP controller was also evaluated (Figure 5B). When the follower handle contacted a hard surface, a position error of up to 14 ° occurred between the both handles, as the leader handle continued to move slightly before resistance was perceived. However, once this error was reached, the leader handle could no longer move forward, effectively rendering contact with a hard surface. Some oscillations could still occur during contact due to system delay.

### 3.2 PF controller

#### 3.2.1 Virtual follower environment

**FIGURE 6:**
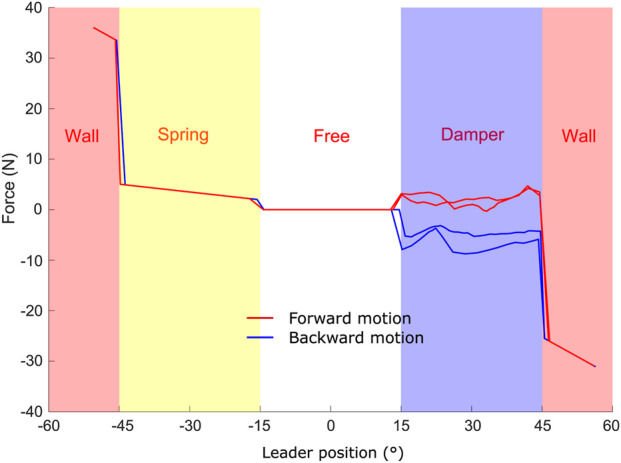
Feedback force rendered by the environment on the leader device as a function of the leader handle position. For clarity, the test was repeated twice using forward and backward motion across the entire range of movement.

The PF controller was evaluated in two stages. Initially, it was tested using a single physical unit as the master, while the follower was virtually simulated in MATLAB. This approach enabled the validation of the controller independently of the follower’s sensor for force feedback. The virtual follower tracked the position of the physical master device and interacted with various simulated environmental force profiles, as illustrated in Figure 6, with the initial position set at the center of the gear system. The simulated environment comprised four distinct regions: (i) Wall regions (from −60 ° to −45 ° and 45 ° to 60 ° ), representing rigid boundaries that exerted a force proportional to displacement. The maximum force was constrained by the motor’s torque limit (307.8 g ⋅ cm), corresponding to approximately 23 N/ ° of follower displacement, which was used as the wall stiffness. As shown in Figure 6, the follower entered the wall beyond ±45° , indicating that the user applied forces exceeding the motor’s maximum output. (ii) A spring region (−45 ° to −15 ° ), where the follower experienced a linearly increasing force with position, with a slope set to 0.1 N/ ° . (iii) A free space region (−15 ° to 15 ° ), a neutral zone without any resistance. (iv) A damper region (15 ° to 45 ° ), where the follower encountered a damping force proportional to its velocity, with a damping coefficient of 1.5 Ns/ ° . As shown in Figure 6, despite its low-cost and relatively simple hardware and software, the system was capable of rendering various virtual environments, similar to the Hapkits, while employing a different hardware and software architecture. In particular, the Telekit used a gear system for torque transmission and relied on MATLAB for data management.

#### 3.2.2 Real follower environment

In a second stage, the performance of the PF controller was evaluated under real-world conditions, meaning that the follower unit interacted physically with various environments. Specifically, a sponge, a soft spring, and a piece of hard metal were used to test these interactions, enabling realistic force feedback. The perceived stiffness for each environment was calculated as the ratio between the force measured by the follower’s force sensor (transmitted to the leader) and the displacement of the leader’s handle. Because this metric was derived from measured physical quantities, it did not depend on user judgment. The perceived stiffness values were compared to the actual stiffness of each material, which was measured independently by applying known forces directly to the objects and recording their deformation outside the teleoperation setup.

**FIGURE 7:**
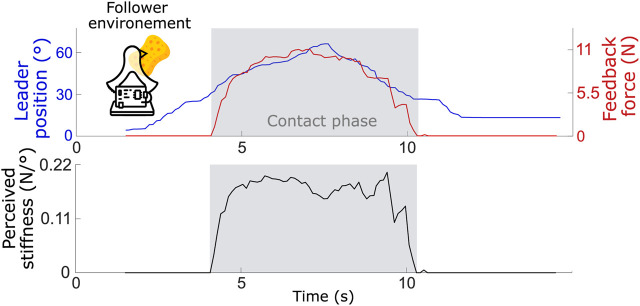
Leader handle position, feedback force and user perceived stiffness for one test (average of 10 tests is of 0.16 ± 0.07 N/ ° ) when the follower makes contact with a sponge.

**FIGURE 8:**
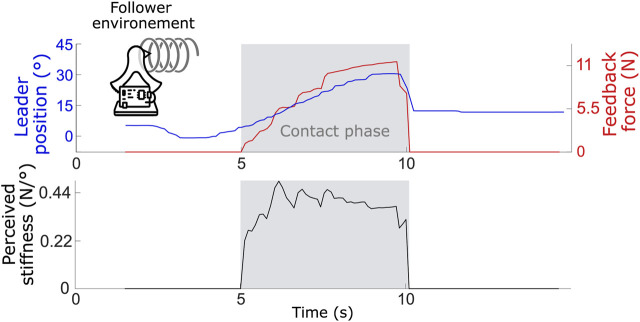
Leader handle position, feedback force and user perceived stiffness for one test (average of 10 tests is 0.42 ± 0.1 N/ ° ) when the follower makes contact with a spring of 0.52 N/mm (equivalent to 0.67 N/ ° ).

For each environment, 10 tests were performed. When the follower made contact with a sponge (see Figure 7 for an example test), the perceived stiffness had an average value of 0.16 ± 0.07 N/ ° , or 0.12 N/mm. The reported variability corresponds to the standard deviation across the repeated trials. When the follower interacted with a spring with a known stiffness of 0.52 N/mm (equivalent to 0.67 N/ ° ) (see Figure 8 for an example test), the average perceived stiffness reported by the user was 0.42 ± 0.1 N/ ° .

**FIGURE 9:**
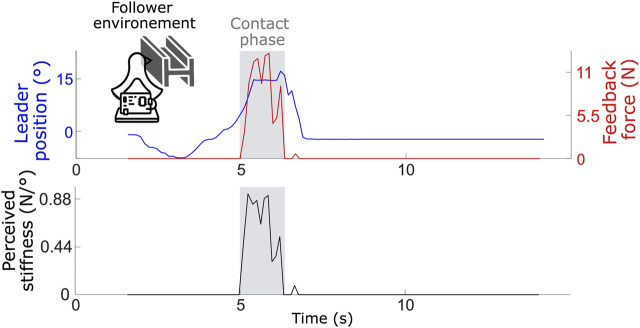
Leader handle position, feedback force and user perceived stiffness for one test (average of 10 tests is 0.52 ± 0.13 N/ ° ) when the follower makes contact with a hard metallic piece.

This discrepancy between the perceived and actual stiffness could be attributed to factors such as the system compliance, sensor noise, and possible time delays, which could dampen the perceived force. Finally, when the follower came into contact with a hard metal surface (see Figure 9 for an example test), the user perceived an average stiffness of 0.52 ± 0.13 N/ ° . However, as shown in Figure 9, the follower experienced oscillations upon contact, similar to those observed when the PP controller was used (Figure 5B). These oscillations were reflected in the force feedback, making the haptic sensation less stable and less reliable. These oscillations also limited the ability of the user, and therefore the follower unit, to apply additional force onto the hard surface. Despite this, the system demonstrated the capability to convey three distinct levels of environmental stiffness to the user: soft (the sponge), medium (the spring), and hard (a metal surface), highlighting the effectiveness of the system and the implemented PF controller in representing gradations in contact stiffness.

## 4 Discussion

Regarding the design of the Telekits, the transition from friction- or capstan-based transmission mechanisms to a spur gear system was primarily motivated by the need for robustness under repeated student use. However, spur gears introduce backlash and reduced smoothness due to manufacturing tolerances, which likely contributes to the oscillations observed during contact experiments. This backlash constitutes one of the main mechanical limitations of the current prototype, as it introduces small dead zones in the transmission and reduces motion transparency during rapid direction changes. Possible improvements consist of using higher-precision machined gears and/or implementing a helical or herringbone gear, which engage gradually (not suddenly like spur gears), resulting in smoother motion. Such solutions could reduce backlash and improve the perceived smoothness of the teleoperation interaction. As for the force sensing mechanism, the chosen force-sensitive resistor was selected for its low cost and wide availability. Its placement was designed to minimize modifications to the original Hapkit structure, allowing the Telekit to function as a modular add-on. However, based on the results obtained with the PF controller during real-environment interactions, the current force sensing setup could not accurately render physical contact. While the system successfully conveyed qualitative differences in environment stiffness, which is promising for educational or demonstration purposes, the quantitative fidelity of the feedback remains limited. Improvements could be achieved by reconsidering both the type and placement of the force sensor. For example, integrating a load cell inside the handle, connecting its upper and lower parts, could provide more direct and reliable force measurements.

Regarding the communication and control system, the implemented setup exhibited delays ranging from 140 ms to 280 ms. Across both PP and PF control, the data show that communication delay is the primary factor degrading tracking accuracy and contact stability. Although the measured communication delay at the data transmission level is only a few milliseconds, the effective system delay is significantly larger due to the accumulation of sensor acquisition time, MATLAB processing, control loop execution, and motor actuation dynamics. Low-frequency motions are less affected because friction-related effects dominate, while high-frequency or contact interactions amplify the impact of delay, leading to oscillations. Therefore, further refinement of the control strategy could enhance system stability. For instance, implementing adaptive damping or predictive control techniques could enhance contact stability.

Regarding the PF controller characterization, although the stiffness estimation presented in this work is fully quantitative and derived from sensor-based measurements, the current evaluation does not yet include psychophysical assessments that would capture how users perceive and discriminate stiffness through the teleoperation interface. In particular, tests such as just-noticeable-difference (JND) measurements, user-level discrimination trials, or confusion analyses were not conducted. As this paper primarily focuses on the design and control of the Telekit, psychophysical evaluation falls outside the current scope. Such assessments will be carried out in future work, and could also be incorporated as part of student assignments within the haptics course.

The Telekits were introduced to 27 students, working in groups of 3 who had no prior hands-on experience with teleoperation, having only studied the theoretical background. Some of the students had a foundation in control theory. The intended learning outcome of the assignment was for students to design and implement both PP and PF controllers for the 1-DoF teleoperation setup. The complete hardware and software setup was provided, excluding only the controller component. The students were tasked with implementing both PP and PF controllers, allowing them to experiment with PD controller parameters and observe their effects on the teleoperated system. Additionally, they were asked to implement a delay compensation strategy and evaluate its performance on the setup. Each group had access to the Telekits for up to 5 hours of hands-on testing. All groups successfully completed the assignment. Although a full educational study was not conducted, preliminary feedback was collected during the course in which the Telekits were used. Five student groups completed a short survey, with 20% reporting that the Telekit improved their understanding of teleoperation “a great deal,” 60% “quite a bit,” and 20% “a little.” During the lab sessions, students also reported concrete observations that aligned with the system’s technical behavior: increased oscillations at higher motion frequencies were clearly perceived and soft, medium, and hard environments could be distinguished. These experiences illustrate how the platform helps bridge theoretical concepts (e.g., delay, damping, stiffness) and hands-on understanding. The present educational evaluation is preliminary, and a more extensive study will be performed in future work.

Compared with existing educational teleoperation platforms, the Telekit bridges the gap between virtual teaching tools and expensive research-grade systems. Virtual environments are low-cost but lack physical interaction, whereas commercial haptic devices (e.g., Geomagic Touch) provide high performance but are costly and complex for classroom use. The Telekit provides a low-cost physical platform enabling both motion synchronization and force feedback. Compared to the original Hapkit, which focuses on single-device haptics, the Telekit enables bilateral leader–follower teleoperation experiments.

In conclusion, the proposed Telekit platform provides a low-cost and accessible system for hands-on learning in bilateral teleoperation, enabling students to bridge theoretical concepts with practical implementation. Built on the Hapkit platform and extended with bilateral communication and force sensing, the system supports both Position–Position and Position–Force control strategies and allows students to experiment with motion synchronization and force-feedback interactions in both virtual and real environments. The platform was successfully deployed in a teaching context, where students with no prior teleoperation experience implemented and tuned control strategies and directly observed the effects of delay, damping, and environmental stiffness during hands-on experiments. Although the system demonstrates the feasibility of a low-cost bilateral teleoperation platform for education, several limitations remain, including mechanical backlash from the gear transmission, limited force sensing accuracy, and system delays that affect high-frequency tracking and contact stability. Future work will therefore focus on improving the mechanical transmission, enhancing force sensing accuracy, and optimizing the communication and control architecture to improve responsiveness and stability. Additional studies will also investigate the educational impact of the platform through more systematic student evaluations.

## Data Availability

The raw data supporting the conclusions of this article will be made available by the authors upon request.

## References

[B1] Beerens R. Heck D. Saccon A. Nijmeijer H. (2019). The effect of controller design on delayed bilateral teleoperation performance: an experimental comparison. IEEE Trans. Control Syst. Technol. 28, 1727–1740. 10.1109/tcst.2019.2923703

[B2] Candelas F. Torres F. Ortiz F. Gil P. Pomares J. Puente S. (2003). Teaching and learning robotics with internet teleoperation 3, 1827–1831.

[B3] Dijkstra I. van de Kant M. Wildenbeest J. Hoevenaars T. (2014). Tu delft hapkit – 1 dof telemanipulator.

[B4] Hofstein A. Lunetta V. N. (2004). The laboratory in science education: foundations for the twenty-first century. Sci. Education 88, 28–54. 10.1002/sce.10106

[B5] Hokayem P. F. Spong M. W. (2006). Bilateral teleoperation: an historical survey. Automatica 42, 2035–2057. 10.1016/j.automatica.2006.06.027

[B6] Kaarlela T. Arnarson H. Pitkäaho T. Shu B. Solvang B. Pieskä S. (2022). Common educational teleoperation platform for robotics utilizing digital twins. Machines 10, 577. 10.3390/machines10070577

[B7] Koenig A. y Baena F. R. Secoli R. (2021). Gesture-based teleoperated grasping for educational robotics, 222–228.

[B8] Lee D. Spong M. W. (2006). Passive bilateral teleoperation with constant time delay. IEEE Transactions Robotics 22, 269–281. 10.1109/tro.2005.862037

[B9] Marinho M. M. Lin H.-C. Zhao J. (2023). Umirobot: An open-{Software, Hardware} low-cost robotic manipulator for education, 4464–4471.

[B10] Martinez M. O. Nunez C. M. Liao T. Morimoto T. K. Okamura A. M. (2019). Evolution and analysis of hapkit: an open-source haptic device for educational applications. IEEE Transactions Haptics 13, 354–367. 10.1109/TOH.2019.2948609 31634847

[B11] Marwedel P. (2021). Embedded system design: embedded systems foundations of cyber-physical systems, and the internet of things. Springer Nature.

[B12] Millnert V. (2014). Quality-latency trade-off in bilateral teleoperation

[B13] Nuño E. Basañez L. Ortega R. Spong M. W. (2009). Position tracking for non-linear teleoperators with variable time delay. Int. J. Robotics Res. 28, 895–910. 10.1177/0278364908099461

[B14] Nuño E. Basañez L. Ortega R. (2011). Passivity-based control for bilateral teleoperation: a tutorial. Automatica 47, 485–495. 10.1016/j.automatica.2011.01.004

[B15] Okamura A. M. Blikstein P. Morimoto T. Davis R. (2014). Hapkit: an open-hardware haptic device for education.

[B16] Pala M. Lorencik D. Sincak P. (2012). Towards the robotic teleoperation systems in education, 241–246.

[B17] Rojko A. Hercog D. (2015). Motion control and teleoperation challenge. 001662–001667.

[B18] Yang X. Chen Q. Petriu D. C. Petriu E. M. (2004). Internet-based teleoperation of a robot manipulator for education, 7–11.

[B19] Yang Y. Hua C. Guan X. (2015). Finite time control design for bilateral teleoperation system with position synchronization error constrained. IEEE Transactions Cybernetics 46, 609–619. 10.1109/TCYB.2015.2410785 25823053

